# Data curation to support toxicity assessments using the Integrated Chemical Environment

**DOI:** 10.3389/ftox.2022.987848

**Published:** 2022-11-03

**Authors:** Amber B. Daniel, Neepa Choksi, Jaleh Abedini, Shannon Bell, Patricia Ceger, Bethany Cook, Agnes L. Karmaus, John Rooney, Kimberly T. To, David Allen, Nicole Kleinstreuer

**Affiliations:** ^1^ Inotiv, Research Triangle Park, NC, United States; ^2^ NIH/NIEHS/DTT/NICEATM, Research Triangle Park, NC, United States

**Keywords:** data curation, data harmonization, FAIR, toxicology, non-animal methods

## Abstract

Humans are exposed to large numbers of chemicals during their daily activities. To assess and understand potential health impacts of chemical exposure, investigators and regulators need access to reliable toxicity data. In particular, reliable toxicity data for a wide range of chemistries are needed to support development of new approach methodologies (NAMs) such as computational models, which offer increased throughput relative to traditional approaches and reduce or replace animal use. NAMs development and evaluation require chemically diverse data sets that are typically constructed by incorporating results from multiple studies into a single, integrated view; however, integrating data is not always a straightforward task. Primary study sources often vary in the way data are organized and reported. Metadata and information needed to support interoperability and provide context are often lacking, which necessitates literature research on the assay prior to attempting data integration. The Integrated Chemical Environment (ICE) was developed to support the development, evaluation, and application of NAMs. ICE provides curated toxicity data and computational tools to integrate and explore available information, thus facilitating knowledge discovery and interoperability. This paper describes the data curation workflow for integrating data into ICE. Data destined for ICE undergo rigorous harmonization, standardization, and formatting processes using both automated and manual expert-driven approaches. These processes improve the utility of the data for diverse analyses and facilitate application within ICE or a user’s external workflow while preserving data integrity and context. ICE data curation provides the structure, reliability, and accessibility needed for data to support chemical assessments.

## 1 Introduction

Humans encounter numerous chemicals through a variety of activities, including food consumption, use of consumer products, and work in manufacturing or agricultural settings. Understanding how these encounters impact human and environmental health can be challenging. As the numbers and complexities of human chemical exposures increase, traditional animal-based chemical safety assessment approaches are insufficient to provide timely and human-health relevant information needed for regulators to ensure that new products and applications do not cause unintended harm. In recent years, researchers, test method developers, and regulators have proposed increased use of *in vitro* systems and computational methods to decrease reliance on animal methods (NRC, 2007). Adoption of such new approach methodologies (NAMs) would increase throughput and decrease costs of toxicity testing while improving human-health relevance (ICCVAM, 2018).

Development and acceptance of NAMs requires that the method’s performance be evaluated, usually by benchmarking performance against historical animal-based toxicity studies, which typically investigate a limited number of chemicals or doses. To facilitate the evaluation of NAMs and increase confidence in their fit-for-purpose use, a broad range of appropriate data is required. Integrating data from multiple small-scale studies can facilitate formation of benchmarks for NAMs concordance evaluations.

The National Toxicology Program Interagency Center for the Evaluation of Alternative Toxicological Methods (NICEATM) is charged with evaluating and promoting methods that replace, reduce, or refine the use of animals for chemical safety testing. Because development and evaluation of NAMs as alternatives to animal-based safety tests requires reliable data, data curation is paramount to NICEATM’s mission. To aid test method development and build confidence in these approaches, NICEATM developed the Integrated Chemical Environment^1^ (ICE) to support the availability and contextualization of relevant data.

ICE provides free, unrestricted access to a large collection of curated *in vivo*, *in vitro* and *in silico* data as well as computational tools to support the development and evaluation of NAMs (Bell et al., 2017; Bell et al., 2020; Abedini et al., 2021). Herein we describe the curation processes used to prepare data for inclusion in ICE, including key features intended to improve the interoperability of ICE data. While the processes are described in the context of ICE, the concepts are applicable to similar resources that aim to make data accessible and understandable for a diverse audience.

## 2 Creation of a data resource to support toxicity assessments

Creation of a data resource to support toxicity assessments involves integration and organization of data collected from a number of sources. Most traditional toxicity data are obtained from *in vivo* animal studies that test one or two chemicals and evaluate specific outcomes of exposure such as developmental impacts or increased cancer incidence. Comparison and integration of these data to develop or evaluate NAMs is facilitated by compiling them into a single large resource. However, creating such a resource can be challenging, in part because experimental findings are not generally reported consistently across studies. For example, studies may report the same effect using synonymous terms (e.g., swelling vs. edema) or in different units (e.g., mM vs. ug/ml). Data may also be reported inconsistently with respect to presentation (e.g., tabular vs. graphical) or the level of detail used to describe the method. Furthermore, the quality of metadata surrounding a data point, such as information about the research method used and the chemical tested, can vary dramatically since there are no widely adopted common standards for reporting such details, and standards that do exist (e.g., MIATE^2^, OECD^3^) are infrequently applied.

To provide a resource that is useful for evaluation and application of NAMs, primary toxicity data must have sufficient context that allows users to infer the potential impacts of chemical exposures. Providing access to curated toxicity data with standardized study information enables investigators and regulators to use collective results from multiple studies to assess the potential impact of chemical exposure to human health.

### 2.1 Identification of user needs

The first steps in creating a data resource for supporting toxicity assessments are to identify prospective stakeholders and users and to characterize their needs. These activities help guide the selection of appropriate data and associated metadata or experimental details to be extracted as well as the appropriate formatting for the data.

ICE was developed to support a wide range of NICEATM stakeholders, including regulatory authorities, chemical manufacturers, academic researchers, and test method developers (Bell et al., 2017). Each of these stakeholder groups has unique goals and differing levels of needs that can also vary within the respective groups. For example, a regulator performing a risk assessment may want to compile a broad array of toxicity data for a specific chemical, while a chemical manufacturer developing a new formulation may want to compare the possible toxicological profiles of structurally similar compounds. Academic researchers may be interested in hypothesis generation for adverse effects of various chemicals, and a test method developer may need a validation data set for benchmarking a new *in vitro* approach or training data to develop a new computational model.

Based on such use cases, the following were established as required characteristics for ICE data:

• Data organization keeps primary user needs at the forefront (i.e., data are organized according to regulatory endpoints of interest, also referred to as toxicity endpoints, such as acute lethality).
• Data are findable and interpretable by individuals with varying levels of subject matter knowledge.
• Annotation allows users who are not subject matter experts to identify relevant data.
• Sufficient metadata, when available, to allow assessment of key study parameters for a given endpoint and to aid users in understanding how data were generated.
• Structure allows users to find and compare data from multiple toxicity endpoints and study types.
• Terminology is harmonized/standardized to support interoperability.
• Outputs are compatible with different internal and external applications.
• Data are formatted to be both human- and machine-readable.

Meeting these user needs is consistent with ensuring that the data in ICE continue to move toward compliance with FAIR (findable, accessible, interoperable, and reusable) principles (Wilkinson et al., 2016), and fulfill standards set forth by the National Institutes of Health for scientific data resources.

### 2.2 Data curation

**FIGURE 1:**
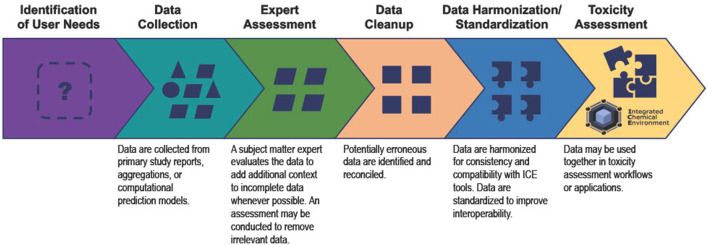
The ICE data curation process enables ICE data to better support user needs. Data collected from various sources lack interoperability and harmonization. NICEATM curates ICE data so that meaning is preserved, and users can access and compare data for toxicity assessments.

Reliable models and decisions require robust and reproducible data. However, there are few existing conventions addressing how to identify and curate these data. The processes NICEATM follows to curate data to accommodate the user needs listed above are outlined in Figure 1 and detailed in the following sections.

#### 2.2.1 Data collection

Incorporation of data into ICE begins with data collection. Data needed to support chemical assessment using NAMs and NAMs development come from diverse sources requiring different curation approaches. While there are some exceptions, ICE *in vivo* and *in vitro* data are generally collected from three types of sources: individual primary study reports (and/or literature review efforts encompassing such studies), large aggregations of summarized data from repositories such as PubChem, and computational prediction models. Primary study reports generally provide an adequate level of metadata to assess whether the data are suitable for use in evaluations of NAMs. Large data aggregations, however, may have limited metadata, which makes evaluation of study quality difficult. Computational model predictions are evaluated for suitability and reliability by assessing the model documentation, applicability domain or uncertainty parameters, and training data, when available. Additionally, ICE contains curated high-throughput screening (cHTS) data derived from the Tox21 Consortium^4^ retrieved from the U.S. Environmental Protection Agency’s (EPA) InvitroDB (EPA, 2021b). ICE curators consider the specific characteristics of data to curate and apply quality flags to optimize their usability.

#### 2.2.2 Expert assessment

In the next step of ICE data curation, subject matter experts evaluate data relevant to the toxicity endpoint(s) within their purview. They identify which data are most relevant to accommodate ICE user needs and provide additional context for incomplete data whenever possible. In some cases, the subject matter expert may select the subset of data that is most useful to stakeholders for inclusion in ICE. For example, the rodent uterotrophic assay data in ICE are from a study that compiled data to benchmark the performance of novel *in vitro* methods for identification of chemicals that may disrupt estrogen receptor signaling (Browne et al., 2015; Kleinstreuer et al., 2016). The compiled study data were assessed to determine adherence to six criteria based on regulatory guidelines for the uterotrophic assay (OECD, 2007; EPA, 2011). Only those data meeting all criteria are included in ICE. Even though this assessment reduced the number of chemicals represented, the alignment of the resulting data set with test method standards described in the aforementioned regulatory guidelines increased its utility for evaluation of NAMs that measure estrogenic activity and therefore improved the overall usefulness for ICE primary users.

Subject matter experts may also identify errors or draw on their specialized knowledge to provide additional context for data. This is most beneficial for curating data from large aggregations where metadata are often lacking. For example, if the species is not reported for a local lymph node assay result, it is rational to conclude that the species must be mouse based on the internationally harmonized test guideline (OECD, 2010). Similarly, if the sex is not included with Hershberger results, an expert familiar with the study protocol could conclude that all test subjects are male (EPA, 2009). Documentation of rationale for such modifications to metadata, along with version control history, are internally maintained for data provenance. Annotated data are provided in ICE to make the information easier to understand for users not familiar with the various toxicity assays and the details of their associated regulatory guidelines.

#### 2.2.3 Data cleanup

The next step in the ICE curation process is to identify and address variations in capitalization and spelling, special characters that are incompatible with computational tools for data analyses, typographical errors, and other minor issues that inherently occur when data are collected from diverse sources. ICE data are processed through an automated workflow to reconcile such differences. Data cleanup helps provide a smoother transition to the harmonization and standardization process by resolving inconsistencies. This is particularly true for subsequent automated steps where minor issues (e.g., trailing spaces, carriage returns) can obstruct the workflow.

One of the most important components of curating a reliable data resource is the identification and possible flagging or removal of suspect data points to improve overall fidelity. Since it is not always feasible to examine individual studies, ICE curators use a combination of automated and manual workflows to check data consistency and identify any anomalies (e.g., numeric outlier values) within the data set. These workflows help to identify potential errors, whether from the original source or inadvertently introduced during data collection or expert assessment, for further manual review and verification.

In addition to identifying errors and making data sets more compatible for automated processing, the ICE data cleanup step also provides an opportunity to identify duplicate, identical data points. Unintended duplication of study data can occur when a single test is reported in multiple sources. This may occur if the same data are collected both from a primary study and a review article citing that study, or inadvertent inclusion of read-across data. Duplications of a single data point can cause an inaccurate perception of chemical bioactivity or study reproducibility and therefore should be removed from the curated data set. As an example, *in vivo* skin irritation data in ICE were compiled to characterize the reproducibility of the rabbit skin irritation test (Rooney et al., 2021). The initial data set was generated by querying a large data repository for a defined set of parameters intended to limit information type to experimental results (i.e., read-across predictions were excluded). However, during the data cleaning step, a number of repeated values were flagged as potential read-across predictions. Chemicals represented in the data set were grouped based on structural similarity, and primary sources associated with repeated values within each group were reviewed to verify whether the values were experimental or read-across. Confirmed read-across predictions and results with insufficient information from the primary data source were removed from the data set (Rooney et al., 2021). ICE provides front-end annotations of the original reference citations and, when available, Digital Object Identifiers and PubMed^®^ reference numbers associated with each result to help users differentiate experiments and locate publicly available data sources if required for further evaluations.

#### 2.2.4 Data harmonization/standardization

The final step in the ICE data curation process is data harmonization and standardization. Since data for inclusion in ICE are collated from a variety of sources and no universally accepted format for reporting results exists, initial data sets vary in their use of chemical identifiers, units of measurement, terminologies, and metadata. Homogenizing these disparate elements by applying a common structure enables users to access all available information and creates a straightforward path to data discovery and analysis. Harmonization is facilitated by use of a semi-automated workflow customized to improve consistency of capitalization and remove Greek letters, symbols, and special characters. ICE data are harmonized to the extent possible (e.g., harmonization of units), and metadata are standardized across studies by aligning with those used in relevant authoritative standards, such as regulatory guidance materials. This standardization promotes interoperability with external data resources such as the EPA CompTox Chemicals Dashboard (Williams et al., 2017; EPA, 2021a) and enables users to process the data through applications or workflows for toxicity assessments. Further, the cHTS data in ICE are annotated using controlled terminology from the NCI Metathesaurus^5^. This enables mapping of assay mechanistic targets to associated modes of action, thereby helping connect assays to relevant toxicity endpoints (Bell et al., 2020).

Chemical identifiers are harmonized to allow users to easily access all data available for all toxicity endpoints within ICE in a search query for a specific chemical. At data collection, chemical identifiers are typically extracted precisely as reported in the primary source. For example, four separate studies may identify a test substance as “bisphenol A,” “BPA,” “4,4′-isopropylidenediphenol,” and Chemical Abstracts Service Registry Number (CASRN) “80-05-7.” If sufficient detail is provided in the references, the data curator can confidently conclude that the test substances are structurally identical. To eliminate ambiguity caused by differing identifiers and synonyms, chemical identifiers in ICE are standardized to align with those in the EPA Chemicals Dashboard (Williams et al., 2017; EPA, 2021a). Each chemical in ICE is identified by the chemical name, CASRN, EPA Distributed Structure-Searchable Toxicity substance identifier (EPA, 2015) and SMILES string retrieved from the Dashboard. Users can easily access all available data through the ICE web-browser Search tool or the ICE Search Representational State Transfer Application Programming Interface^6^.

Similarly, units of measurement frequently vary between sources and require standardization to facilitate interoperability needed to apply data in NAM workflows. While there are no mandates for reporting test results using specific units of measurement, some regulatory guidelines for animal or non-animal test methods may specify a preferred unit. In these cases, ICE data are standardized by converting units, when possible, to align with the applicable guidance document(s). Only converted values are accessible through ICE tools; however, reported values and conversion information are provided in full data set downloads^7^ whenever non-scale conversions (i.e., those that involve a conversion factor) are applied.

## 3 Discussion

As humans are exposed to increasing numbers of chemicals during daily activities, the need grows stronger to expedite chemical safety assessments. NAMs offer the potential to increase throughput and decrease costs of these assessments relative to traditional animal approaches. However, development, application, and regulatory acceptance of NAMs can be hindered by a lack of toxicity data that are suitable for method evaluation. Additionally, as new mechanistic *in vitro* methods are developed, understanding how these methods relate to traditional *in vivo* toxicity test endpoints can be difficult for some that are less familiar with the field. Thus, resources that integrate curated data from multiple studies into robust, well-annotated, and computer-readable data sets are essential to facilitating chemical assessments and adoption of NAMs. NICEATM’s role in supporting the U.S. federal government’s efforts toward alternatives to animal testing puts it in a unique position to address these data needs. It can be time-consuming to review data and determine the level of curation needed to integrate multiple studies into a standardized data set, particularly if the metadata are lacking. However, applying a robust curation strategy across data sets is necessary to add additional context needed to facilitate chemical assessments and adoption of NAMs. Collectively, the curation, harmonization, and standardization efforts that NICEATM conducts to prepare and maintain data in ICE improve the usability of these data, giving stakeholders easier access to and higher confidence in the data. As an added benefit, ICE offers tools that can leverage these data to enable further stakeholder engagement with computational toxicology workflows and *in silico* approaches for characterizing chemical bioactivity.

## Data Availability

The original contributions presented in the study are included in the article/supplementary material, further inquiries can be directed to the corresponding author.

## Footnotes

1
https://ice.ntp.niehs.nih.gov/.

2
https://github.com/zacharewskilab/MIATE/.

3
https://www.oecd.org/ehs/templates/.

4
https://tox21.gov/.

5
https://ncim.nci.nih.gov/ncimbrowser/.

6
https://ice.ntp.niehs.nih.gov/About/.

7
https://ice.ntp.niehs.nih.gov/DATASETDESCRIPTION/.

## Abbreviations

CASRN, Chemical Abstracts Service Registry Number; cHTS, curated high-throughput screening; EPA, U.S. Environmental Protection Agency; FAIR, findable, accessible, interoperable, and reusable; ICE, Integrated Chemical Environment; NAMs, new approach methodologies; NICEATM, National Toxicology Program Interagency Center for the Evaluation of Alternative Toxicological Methods.
